# Configurational Paths of Depression and Anxiety Symptoms in Ovarian Cancer Patients: A Fuzzy‐Set Qualitative Comparative Analysis

**DOI:** 10.1155/da/6451069

**Published:** 2026-07-18

**Authors:** Chaoying Ma, Jiani He, Li Liu, Zhichen Liu, Yajing Wang, Hui Guo, Yiqun Xiao, Meizhu Pan, Yuli Song, Yi Zhang, Hui Wu

**Affiliations:** ^1^ Department of Social Medicine, China Medical University, Shenyang, China, cmu.edu.tw; ^2^ Department of Gynecology, The First Affiliated Hospital of China Medical University, Shenyang, China, cmu.edu.cn

**Keywords:** anxiety symptoms, configuration, depression symptoms, fuzzy-set qualitative comparative analysis, ovarian cancer

## Abstract

**Background:**

Ovarian cancer is one of the most common gynecological cancers in women. Its diagnosis and treatment often cause significant psychological distress, mainly reflected in depression and anxiety, which arise from multiple interacting factors. This study, grounded in Lazarus’s Stress and Coping Model and from the perspective of set theory, aimed to investigate the correlates and configurational pathways of high and low levels of depression and anxiety symptoms in ovarian cancer patients by integrating stress sources, coping styles, and coping resources.

**Methods:**

The study recruited ovarian cancer patients from a tertiary general hospital in Shenyang, China. The analysis included 169 ovarian cancer patients. The configurational factors included self‐perceived burden (SPB), positive and negative coping styles, self‐esteem, and general self‐efficacy. Multiple linear regression and fuzzy‐set qualitative comparative analysis (fsQCA) were used to explore the factors and configurational pathways related to depression and anxiety symptoms.

**Results:**

The fsQCA identified six configurational pathways linked to high and low levels of depression symptoms. Similarly, the fsQCA identified two configurations linked to high anxiety symptoms and four configurations related to low anxiety symptoms. Moreover, the configurations associated with low psychological symptoms did not stand in opposition to those associated with high psychological symptoms. Substitutability and complementarity were observed among the various elements in the configuration.

**Conclusion:**

The study generated multiple configurational pathways for depression and anxiety symptoms in ovarian cancer patients by examining the combined effects of stress sources, coping styles, and coping resources. The configuration allows caregivers and medical staff to implement targeted measures to reduce burden perception and negative coping, enhance positive coping and self‐esteem considering their substitutability and complementarity.

## 1. Introduction

Globally, 324,398 new ovarian cancer cases were reported in 2022, comprising 1.6% of all cancer cases. Additionally, 206,839 individuals succumbed to ovarian cancer, which constituted 2.1% of all cancer‐related deaths [1]. As a common malignant tumor, ovarian cancer is notable for its high incidence and mortality rates within the female reproductive system [2]. Although treatment advancements have been made, ovarian cancer continues to be the deadliest among all gynecologic cancers [3]. Due to its insidious onset, ovarian cancer has an age‐standardized 5‐year relative survival rate of merely 39.6% [4]. Psychological distress is frequently intensified by the diagnosis and treatment of ovarian cancer, which adversely affects patients’ quality of life. Research indicates that compared to individuals with other malignancies, women with ovarian cancer tend to endure higher levels of psychosocial distress [5].

Ovarian cancer patients frequently experience depression and anxiety as major psychological comorbidities. A meta‐analysis revealed that 27% of ovarian cancer patients experience depression symptoms, while 33% suffer from anxiety [6]. Compared to women without an ovarian cancer diagnosis, the risk of clinically significant depression and anxiety in ovarian cancer patients is approximately two and four times greater [5]. Meraner et al. [7] examined the progression of physical and psychosocial symptoms in ovarian cancer patients following chemotherapy and revealed that these patients experienced significant fatigue, along with elevated levels of anxiety and depression following surgery. The presence of depression and anxiety symptoms among ovarian cancer patients may affect treatment adherence, exacerbate physical discomfort, and lead to various unpredictable adverse outcomes [6].

The intensity of depression and anxiety in ovarian cancer patients stems from a multifactorial network involving both direct and mediated pathways. Based on Lazarus and Folkman’s transactional model of stress and coping, the stress response can be parsed into four interrelated elements: exposure to stressors, the processes of cognitive evaluation, the coping responses selected, and the subsequent consequences [8]. Stress arises when an individual perceives that the external and internal stimuli exceed their coping ability and available resources. This disruption of the individual’s internal equilibrium leads to the experience of stress. Cognitive appraisal is the process through which a person determines a stressor’s relevance and potential impact on the self. It involves identifying and interpreting the stressor and evaluating one’s coping capacities and available resources. The outcomes of coping, in turn, cascade across domains of well‐being, shaping physical and mental health as well as social functioning.

Ovarian cancer patients endure significant physical suffering, financial strain, and psychological distress, and the caregiving from their family members often leads to the perception of being a burden. The phenomenon known as self‐perceived burden (SPB) arises from empathetic concerns where patients fear that their illness and caregiving needs place a substantial burden on their caregivers [9]. As a result, they may experience a range of negative emotions, including depression, anxiety, guilt, and self‐blame [10]. Severe levels of SPB have been linked to suicidal ideation, representing a critical danger to patients’ well‐being [11, 12].

Framing SPB as a stressor leads individuals with ovarian cancer to appraise their coping efficacy—that is, both the strategies they can deploy and the resources available to them. Coping involves cognitive and behavioral efforts within the person–environment interaction to handle internal and external demands perceived as overwhelming one’s resources [13]. Coping strategies such as distancing, humor, reframing or reappraising situations in positive ways, and actively seeking internal and external resources are generally considered adaptive, and strategies such as escape‐avoidance, denial, daydreaming, and blaming others are generally considered maladaptive [14]. Although these coping strategies are diverse and complex, they can be divided into positive and negative coping styles based on their commonalities. The two coping styles are easily observed in real life and understood by people [15]. Evidence from longitudinal studies indicates that individuals who adopt positive coping strategies tend to increase general self‐efficacy, reduce depression and anxiety following an ovarian cancer diagnosis, and improve their quality of life [16, 17].

Among individuals with ovarian cancer, higher levels of self‐esteem and generalized self‐efficacy function as beneficial psychological assets that facilitate stress coping and are linked to improvements in quality of life [18–20]. Self‐esteem refers to people’s overall attitude toward the self: how they judge and evaluate themselves and give themselves value, worth, and respect as human beings [21]. Self‐esteem is vital in reestablishing social roles and functions among cancer patients [22]. Elevated self‐esteem has been shown to facilitate more effective coping with cancer‐related and treatment‐induced stress by facilitating positive psychosocial responses, social support utilization, successful personal control, and well‐being [23]. An individual’s conviction in managing various stressors and challenges is conceptualized as general self‐efficacy [24]. According to the self‐efficacy theory [25], self‐efficacy guides the selection of behaviors and the intensity of effort when confronting obstacles or stressors. Accordingly, general self‐efficacy could play a key role in appraisal and coping processes for threatening situations that might result in different physical and mental symptoms.

Over the years, existing research examining psychological distress in cancer patients has primarily employed net effect analysis as the main analytical approach [26, 27]. Like previous studies on the influencing factors of depression and anxiety in ovarian cancer patients [28–30], traditional net effect analyses such as regression analysis and structural equation modeling focus on testing the independent contribution of each variable to the outcomes. However, the manifestation of depression and anxiety among ovarian cancer patients is shaped by the interplay of various contributing factors, and traditional probability statistical methods that analyze the net effects may not be suitable for explaining such complex relationships. As noted previously, structural equation modeling was not employed because it relies on assumptions grounded in the principle of causal symmetry, which is often unmet in real‐world contexts. For example, the pathways leading to high versus low levels of depression may be fundamentally distinct. Moreover, the stress and coping theory suggests that psychological conditions like depression and anxiety result from the interaction between individual resources and environmental circumstances, not from any single factor. Furthermore, the combined effects of various factors on depression and anxiety symptoms have not been sufficiently discussed. Qualitative comparative analysis (QCA) has been used to clarify the complex pathways of mental health issues [31, 32]. In contrast, QCA is based on the configurational perspective and set theory, captures the complex interdependencies among causal factors, and identifies diverse combinations that jointly lead to specific outcomes (where different configurations and pathways lead to the same outcome). Within the QCA framework, the configurations that produce the presence of an outcome are not the mirror images of those yielding its absence, reflecting causal asymmetry. Among QCA variants, fuzzy‐set QCA (fsQCA), which relies on fuzzy membership scores, is better suited to datasets that mix categorical and continuous variables. By eschewing hard dichotomies of “completely in” or “completely out,” fsQCA offers broader applicability. FsQCA is particularly suitable for examining ongoing psychological determinants of adverse mental health outcomes in cancer patients.

Therefore, this study treated stressors (SPB), coping styles (positive coping and negative coping), and coping resources (self‐esteem and general self‐efficacy) as conditions and applied fsQCA to delineate alternative configurational pathways leading to depression and anxiety symptoms among ovarian cancer patients and examined the role of various elements in the configuration, providing multipathway interventions to improve their mental health.

## 2. Methods

### 2.1. Participants and procedures

This investigation used a cross‐sectional design and enrolled patients with primary ovarian cancer across all stages from a tertiary public general hospital in Shenyang, China. The recruitment of eligible patients was conducted by attending physicians via personal interviews or phone calls. The inclusion criteria contained the following: (1) 18 years and older, (2) pathological diagnosis of primary ovarian cancer, (3) received surgery treatment, adjuvant therapy, or palliative therapy over a month before participation in the study, (4) knowing their own cancer diagnosis, and (5) with normal cognitive functioning and the ability to complete a questionnaire. The exclusion criteria were (1) with a history of mental disorder and (2) with other forms of cancer. QCA can be applied in the research design of small samples and large samples. According to the methodological recommendations for fsQCA, the determination of the sample size should consider the number of conditions that are included in the analysis. Theoretically, if there are k antecedent conditions, then up to 2^k^ possible configurations exist. We included five antecedent conditions; thus, the sample size needed to be greater than 2^5^ = 32.

Between March and June 2021, 214 invited patients consented to participate. After data quality checks, 45 questionnaires were excluded because the item nonresponse exceeded 30% of the survey. In the remaining sample, the missing data proportion for each questionnaire was as follows: 0.12% for the SPBS, 0.39% for the simplified coping style questionnaire (SCSQ), 0.06% for the general self‐efficacy scale (GSES), 0.06% for the Rosenberg self‐esteem scale (RSES), 1.28% for the self‐rating depression scale (SDS), and 0.09% for the self‐rating anxiety scale (SAS). Given the low proportion of missing data, mean imputation was employed to handle the missing values in this study. In the end, 169 patients were included in statistical analysis, with an effective response rate of 79.0%. It is worth emphasizing that because this study adopts a cross‐sectional design, we can only identify the correlation of variables, but no causal inferences can be drawn from the findings.

Ethical approval was obtained from the Ethics Committee of the China Medical University, and all procedures conformed to the ethical guidelines of the Declaration of Helsinki. Prior to enrollment, all participants were fully informed about the study’s objectives, procedures, voluntary nature, and anonymity. For inpatients and outpatients, written informed consent was obtained during face‐to‐face interviews. For discharged patients, interviews were conducted by telephone. In this situation, verbal informed consent was used. Before each telephone interview, our interviewer read the entire informed consent form to the participant and asked for verbal agreement. Upon receiving verbal consent, the interviewer recorded the following information in a consent documentation form: participant ID, date and time of the call, researcher’s name, and a statement that “the consent form was read aloud, and verbal consent was obtained.” The form was signed by the principal investigator and securely stored. In all other respects (content of information provided, voluntariness, and anonymity) of face‐to‐face and telephone interviews, the consent process was identical. Both inpatients and outpatients completed a structured questionnaire through in‐person interviews, while discharged patients did via a telephone interview. Investigators were trained to assist participants in reading the content of the questionnaire without any induction and recording their responses. Depression and anxiety symptoms were assessed using the SDS and the SAS. Participants who scored ≥63 (standard score) on the SDS or ≥60 (standard score) on the SAS were identified as experiencing moderate to severe psychological distress. For participants with moderate to severe distress, we notified the attending physician after the scale assessment. A clinical psychologist from our team provided in‐person or telephone follow‐up and arranged further support or referral as needed. All personal identifiers were removed and replaced with unique study codes. Clinical data extracted from medical records were de‐identified before being released to our research team. No individual identifying information will be disclosed in any publication or report. Paper‐based data files were securely stored in a locked filing cabinet, and electronic data were stored on a password‐protected server within our university. They were accessible only to research team members who had signed confidentiality agreements.

### 2.2. Measures

#### 2.2.1. Demographic and Clinical Characteristics

We collected the demographic characteristics of the participants, including age, marital status, educational attainment, place of residence, and annual household income (in Chinese yuan, CNY). Patients’ clinical profiles were characterized by factors such as onset type, course of disease, cancer stage (according to the 2014 International Federation of Gynecology and Obstetrics [FIGO] staging system for carcinoma of the corpus uteri), surgical approach, whether they had received chemotherapy, whether they had other chronic comorbidities, family history of cancer, and pregnancy history.

#### 2.2.2. SPB

The SPB scale (SPBS) was administered to evaluate patients’ perceived burden on caregivers across physical, emotional, and financial domains [33, 34]. Composed of 10 items, the SPBS employs a five‐point Likert scale from 1 (“never”) to 5 (“always”), producing total scores between 10 and 50. The higher the scores reported, the more severe their SPB. Cronbach’s α of the SPBS was 0.947 in the study.

#### 2.2.3. Coping Styles

The SCSQ, a revision by Xie reflecting the specific traits of the Chinese population [16], is a widely utilized instrument in China for evaluating individuals’ coping styles. The instrument consists of 20 items organized into two dimensions: positive coping and negative coping. Positive coping consisted of items 1 to 12. The Cronbach’s α of the positive coping subscale was 0.868 in the study. Negative coping contains items 13 to 20. The Cronbach’s α of the negative coping subscale was 0.774 in this study. All items of the SCSQ were rated on a four‐point Likert scale and graded from 0 “not use” to 3 “use frequently”. Higher scores on either subscale indicate a stronger propensity toward the corresponding coping style.

#### 2.2.4. General Self‐Efficacy

The GSES was used to assess participants’ overall level of self‐efficacy [24, 35]. The GSES uses a 4‐point Likert format with 10 items, each rated from 1 (“completely incorrect”) to 4 (“completely correct”). Summing all item responses yields a total score ranging from 10 to 40. The higher the scores measured, the stronger their general self‐efficacy. Cronbach’s α of the scale was 0.931 in the study.

#### 2.2.5. Self‐Esteem

The RSES was administered to evaluate patients’ level of self‐esteem [36]. The instrument comprises 10 items with positively worded items and negatively worded items. using a four‐point Likert scale scored from 1 (“very inconsistent”) to 4 (“very consistent”). Summing all item responses yields a total score; the negative items are scored in the reverse way. Higher total scores indicate a greater self‐esteem. In the present sample, the scale showed acceptable internal consistency, with Cronbach’s α = 0.780.

#### 2.2.6. Depression Symptoms

The SDS was used to measure the psychological and physical symptoms of depression [37]. The SDS, developed by Zung [38], the scale comprises 20 items and contains 10 negatively worded items reflecting the presence of symptoms and 10 positively worded items indicating their absence. Items were scored on a four‐point Likert scale from 1 (“a little of the time”) to 4 (“most of the time”), and positive items were coded inversely. The standardized score is obtained by multiplying the total of all 20 items by 1.25 and then taking the integer value of the result. Higher scores indicate more severe depression symptoms. In this study, Cronbach’s α = 0.877.

#### 2.2.7. Anxiety Symptoms

The SAS served as a measure of participants’ anxiety symptom severity [39]. The SAS is developed by Zung [40], which contains a total of 20 items, of which 5 items worded positively are reverse‐scoring items. Responses to each item were recorded on a four‐point Likert scale, with scores ranging from 1 (“a little of the time”) to 4 (“most of the time”). The sum across the 20 items is multiplied by 1.25 to generate a standardized score using the integer part of the result. Higher scores indicate more severe anxiety. In this study, Cronbach’s α = 0.838.

### 2.3. Statistical Analysis

Descriptive, correlational (Pearson), and multiple linear regression analyses were carried out using SPSS 21.0. fsQCA 4.0 was used to explore the configurational paths of depression and anxiety symptoms in ovarian cancer patients. A consistency threshold of 0.8 and a case‐frequency threshold of four were applied, preserving about 84% of cases. For depression as the outcome, the PRI threshold was fixed at 0.7. The PRI for the analysis of anxiety symptoms was set at 0.6. The rows of the truth table corresponding to cases with a PRI below the set threshold are manually changed to 0 to avoid the simultaneous subset relationship between the outcome and negated outcome configurations. Referring to previous studies [41, 42], we adopted adjusting the frequency threshold and calibration anchor points to conduct robustness tests: (i) adjusting the frequency threshold from four to five (retaining about 80% of the cases) and (ii) raising the threshold of calibrated noncrossing anchor points by 2.5%. If the new configuration generated by robustness tests has a clear subset relationship with the original configuration, and the changes in consistency and coverage have not substantially altered the interpretation of the original configuration, then the results can be considered robust [43]. Data analysis was conducted using multiple linear regression and fsQCA. Regression analysis estimates the independent linear effect of each predictor on the outcome while controlling for other variables; a nonsignificant variable in regression indicates no independent statistical predictive power. By contrast, fsQCA focuses on the linkage between conditional configurations and outcomes. A peripheral condition does not exhibit a net effect individually but can combine with other conditions to constitute part of a sufficient configuration for the outcome. As Fiss pointed out [44], variables that are nonsignificant in regression can still serve as necessary conditions or key components of sufficient configurations in QCA.

## 3. Results

### 3.1. Descriptive Statistics

Sixty subjects were aged between 46 and 55 years (35.5%); 89.9% were married or cohabitating. Overall, 34.9% of participants had junior high school education as their highest level attained. Most subjects resided in urban areas (65.1%), 35.5% reported a family annual income below 30,000 yuan, and 36.7% had comorbid chronic diseases. The majority of patients had a history of pregnancy (94.7%), while 19.5% had a family history of cancer. More detailed results are shown in Table 1.

**Table 1:** Demographic and clinical characteristics.

Variables	*n*	%
Age (years)		
≤45	30	17.8
46–55	60	35.5
56–65	57	33.7
≥65	22	13.0
Marital status		
Married/cohabited	152	89.9
Single/divorced/widowed/separated	17	10.1
Educational level		
Primary school or below	28	16.6
Junior high school	59	34.9
Senior high school	38	22.5
Junior college	22	13.0
College or above	22	13.0
Place of residence		
Urban	110	65.1
Rural	59	34.9
Annual household income (CNY)		
<30,000	60	35.5
<80,000	48	28.4
<120,000	47	27.8
≥120,000	14	8.3
Clinical characteristics		
Disease status		
New‐onset	147	87.0
Relapse	22	13.0
Duration of suffering from cancer (years)		
≤1	52	30.8
≤2	34	20.1
≤3	37	21.9
≤4	26	15.4
>4	20	11.8
FIGO stage		
Ⅰ	53	31.4
Ⅱ	40	23.7
Ⅲ	60	35.5
Ⅳ	13	7.7
Not staged	3	1.8
Surgery type		
No operation	2	1.2
Minimally invasive surgery	18	10.7
Laparotomy	149	88.2
Chemotherapy		
No	61	36.1
Yes	108	63.9
Chronic comorbidity		
No	107	63.3
Yes	62	36.7
Pregnancy history		
No	9	5.3
Yes	160	94.7
Family history of cancer		
No	136	80.5
Yes	33	19.5

As shown in Table 2, SPB showed a positive association with depression symptoms, and a negative relationship was observed between depression symptoms and positive coping, self‐esteem, as well as general self‐efficacy. For anxiety symptoms, SPB and negative coping were positively correlated, while positive coping, self‐esteem, and general self‐efficacy were negatively associated with anxiety symptoms.

**Table 2:** The correlations of variables.

Variables	1	2	3	4	5	6
1. SPB	1	—	—	—	—	—
2. PC	−0.123	1	—	—	—	—
3. NC	0.110	0.548 ^∗∗^	1	—	—	—
4. SE	−0.416 ^∗∗^	0.227 ^∗∗^	−0.144	1	—	—
5. GSE	−0.316 ^∗∗^	0.492 ^∗∗^	0.207 ^∗∗^	0.409 ^∗∗^	1	—
6. DS	0.453 ^∗∗^	−0.408 ^∗∗^	0.028	−0.600 ^∗∗^	−0.520 ^∗∗^	1
7. AS	0.447 ^∗∗^	−0.220 ^∗∗^	0.210 ^∗∗^	−0.489 ^∗∗^	−0.292 ^∗∗^	0.801 ^∗∗^

Abbreviations: AS, anxiety symptoms; DS, depression symptoms; GSE, general self‐efficacy; NC, negative coping; PC, positive coping; SE, self‐esteem; SPB, self‐perceived burden.

^∗^ *p* < 0.05.

^∗∗^ *p* < 0.01.

### 3.2. Multiple Regression Models

Table 3 indicates that SPB (*β* = 0.190, *p* < 0.01) and negative coping (*β* = 0.160, *p* < 0.05) exhibited significant positive associations with depression symptoms. In contrast, positive coping (*β* = −0.292, *p* < 0.001), self‐esteem (*β* = −0.347, *p* < 0.001), and general self‐efficacy (*β* = −0.208, *p* < 0.01) demonstrated significant negative associations.

**Table 3:** Regression results of depression symptoms.

Variables	B	SE	*β*	*t*	*p*
Constant	92.360	6.904	—	13.377	<0.001
SPB	0.226	0.072	0.190	3.135	0.002
PC	−0.512	0.131	−0.292	−3.925	<0.001
NC	0.393	0.169	0.160	2.324	0.021
SE	−1.061	0.199	−0.347	−5.321	<0.001
GSE	−0.392	0.127	−0.208	−3.084	0.002

Abbreviations: AS, anxiety symptoms; DS, depression symptoms; GSE, general self‐efficacy; NC, negative coping; PC, positive coping; SE, self‐esteem; SPB, self‐perceived burden.

As shown in Table 4, SPB (*β* = 0.261, *p* < 0.001) and negative coping (*β* = 0.161, *p* < 0.001) showed significant positive associations with anxiety symptoms. Positive coping (*β* = −0.292, *p* < 0.001), and self‐esteem (*β* = −0.347, *p* < 0.001) were negatively associated with anxiety symptoms. The variance inflation factor (VIF) values were all below 5, indicating no problematic multicollinearity.

**Table 4:** Regression results of anxiety symptoms.

Variables	B	SE	*β*	*t*	*p*
Constant	61.847	6.578	—	9.402	<0.001
SPB	0.259	0.069	0.261	3.762	<0.001
PC	−0.412	0.124	−0.282	−3.312	0.001
NC	0.620	0.161	0.303	3.849	<0.001
SE	−0.667	0.190	−0.262	−3.511	0.001
GSE	−0.041	0.121	−0.026	−0.336	0.737

Abbreviations: AS, anxiety symptoms; DS, depression symptoms; GSE, general self‐efficacy; NC, negative coping; PC, positive coping; SE, self‐esteem; SPB, self‐perceived burden.

### 3.3. Calibration

Due to the different distributions of depression and anxiety scores in the sample, we adopted distinct calibration strategies. Aligning with Ragin’s principle that calibration should be guided by the conceptualization of the set, not by mechanical uniformity across outcomes. We calibrated the data with P5 (completely out), P50 (crossover), and P95 (completely in) for SPB, positive coping, negative coping, general self‐efficacy, self‐esteem, and depression symptoms. For anxiety symptoms and the configurational elements, P10, P50, and P90 were directly calibrated as completely out, crossover, and completely in points. Cases with a calibrated membership of exactly 0.5 are borderline and difficult to classify; therefore, they are typically excluded from the analysis. The membership values less than 1 were added to a constant of 0.001. The calibration parameters of configurational elements and depression and anxiety symptoms are shown in Table 5.

**Table 5:** Calibration values.

Statistics	SPB	PC	NC	SE	GSE	DS	AS
M	21.25	22.57	9.41	31.92	27.85	44.53	41.46
SD	10.45	7.07	5.04	4.07	6.59	12.43	10.33
Min	10.00	0.00	0.00	20.00	12.00	25.00	29.00
Max	50.00	36.00	22.00	38.00	40.00	93.00	78.00

Calibration values							

P5	10.00	8.50	1.50	24.00	14.50	28.78	—
P10	10.00	14.00	3.00	26.00	20.00	—	31.25
P50	19.00	23.00	9.00	33.00	29.00	41.31	38.75
P90	38.00	31.00	16.00	37.00	37.00	—	57.50
P95	44.00	34.00	18.50	37.00	40.00	67.50	—

*Note:* P5, 5th percentile, P10, 10th percentile, P50, 50th percentile, P90, 90th percentile, P95, and 95th percentile.

Abbreviations: M, mean; Max, maximum; Min, minimum; SD, standard deviation.

### 3.4. Necessity Analysis

Achieving a consistency value of 0.9 and maintaining adequate coverage are typically regarded as essential criteria for necessity analysis. The findings from the necessity analysis of each single condition for depression symptoms are presented in Table 6. None of the configurational elements served as a necessary condition for high or low depression symptoms. Similarly, the necessity analysis of a single condition for anxiety symptoms is shown in Table 7. Neither high anxiety symptoms nor low anxiety symptoms had necessary conditions.

**Table 6:** The necessary conditions for depression symptoms in ovarian cancer patients.

Condition	High DS		Low DS	
	Consistency	Coverage	Consistency	Coverage
SPB	0.664	0.735	0.514	0.578
~SPB	0.619	0.556	0.764	0.698
PC	0.606	0.684	0.755	0.740
~PC	0.731	0.746	0.576	0.600
NC	0.661	0.656	0.641	0.648
~NC	0.645	0.639	0.660	0.665
SE	0.549	0.537	0.800	0.800
~SE	0.792	0.796	0.535	0.547
GSE	0.569	0.588	0.751	0.791
~GSE	0.798	0.759	0.609	0.589

*Note:* “~” represents a nonset in set theory, a singular condition that manifests through absence.

**Table 7:** The necessary conditions for anxiety symptoms in ovarian cancer patients.

Condition	High AS		Low AS	
	Consistency	Coverage	Consistency	Coverage
SPB	0.680	0.683	0.503	0.571
~SPB	0.573	0.505	0.721	0.718
PC	0.581	0.541	0.651	0.685
~PC	0.662	0.627	0.565	0.604
NC	0.691	0.640	0.573	0.600
~NC	0.566	0.540	0.656	0.706
SE	0.528	0.507	0.714	0.776
~SE	0.767	0.704	0.546	0.567
GSE	0.544	0.552	0.616	0.707
~GSE	0.711	0.621	0.610	0.601

*Note:* “~” represents a nonset in set theory, a singular condition that manifests through absence.

### 3.5. Configurations for Depression Symptoms

In fsQCA, three solution types can be derived: parsimonious, complex, and intermediate. Core conditions are defined as configuration factors appearing in both parsimonious and intermediate solutions as these exert a strong causal effect on the outcome. Peripheral conditions serve a supplementary role and appear exclusively in the intermediate solutions. The study identified three configurational paths for high depression symptoms (HD1, HD2, and HD3) and three configurational paths for low depression symptoms (LD1, LD2a, and LD2b) in ovarian cancer patients. The consistency of each configuration and the overall solution both exceeded 0.89, which indicated good consistency [45]. Thus, 89.4% of the cases included in configurations HD1, HD2, and HD3 exhibited high levels of depression symptoms, while 89.6% of the cases included in configurations LD1, LD2a, and LD2b exhibited low levels of depression symptoms. In addition, the raw coverage of each condition configuration exceeded the standard of 0.25, and the overall coverage reached 0.567 and 0.675, respectively. Configurations HD1, HD2, and HD3 explained 56.7% of the cases for high depression symptoms in the sample, while configurations LD1, LD2a, and LD2b explained 67.5% of the cases for low depression symptoms in the sample, which indicated good coverage. The detailed configurational analysis results are shown in Table 8.

**Table 8:** Configurations for depression symptoms (fsQCA).

Variables	High DS			Low DS		
	HD1	HD2	HD3	LD1	LD2a	LD2b
SPB	●	—	●	—	⨂	⨂
PC	⊗	⊗	—	●	•	⊗
NC	—	●	●	—	•	⊗
SE	⨂	⨂	⨂	●	●	●
GSE	⨂	⨂	⨂	●	—	⊗
Consistency	0.926	0.925	0.924	0.913	0.920	0.918
Raw coverage	0.424	0.395	0.385	0.587	0.454	0.367
Unique coverage	0.106	0.077	0.067	0.106	0.013	0.065
Solution consistency	0.894			0.896		
Solution coverage	0.567			0.675		

*Note:* ●, core condition; •, peripheral condition; ⨂, absent as a core condition; ⊗, absent as a peripheral condition.

Abbreviations: DS, depression symptoms; GSE, general self‐efficacy; NC, negative coping; PC, positive coping; SE, self‐esteem; SPB, self‐perceived burden.

The above fsQCA analysis reveals six configurational pathways leading to high depression symptoms and low depression symptoms. To facilitate clinical understanding and application, the three configurations of High DS are summarized into two core patterns with clinical discriminability, as follows.

The first one is the SPB‐dominant type, which corresponds to configurations HD1 and HD3. Its common feature is the presence of high NC or the absence of PC, with SPB as the core condition. It is characterized by low‐SE and low‐GSE.

Second is the resource‐deficit type, which corresponds to configuration HD2. This pattern is characterized by insufficient personal coping resources accompanied by high NC. Different from the first, SPB is not prominent in this pattern, which suggests that the risk of depression may arise from inadequate internal resources.

The three configurations for low depression symptoms are summarized into two core patterns with clinical discriminability, as follows.

The first one is the resource‐sufficient type, which corresponds to configuration LD1. Three core conditions are present simultaneously: PC, high SE, and high GSE. Patients possess positive coping styles, stable self‐worth, and confidence in their own abilities. Second, it is a low‐SPB and high‐SE dominant type, which corresponds to configurations LD2 and LD3. The core of this pattern lies in the combination of low perceived burden and high SE. Even if NC exists (LD2), PC is absent (LD3), or GSE is relatively low (LD3), low depression is still associated with low SPB and high SE.

### 3.6. Configurations for anxiety Symptoms

The study identified two configurational paths for high anxiety symptoms (HA1a and HA1b) and four configurational paths for low anxiety symptoms (LA1a, LA1b, LA2, and LA3) in ovarian cancer patients. The consistencies of each condition configuration and the overall solution were greater than 0.80, indicating a good consistency. Thus, 85.0% of the cases who met configurations HA1a and HA1b had high anxiety symptoms, while 82.9% of the cases who met configurations LA1a, LA1b, LA2, and LA3 had low anxiety symptoms. The raw coverage of each condition configuration exceeded 0.25, and the overall coverage reached 0.429 and 0.633, respectively, indicating good coverage. Configurations HA1a and HA1b explained 42.9% of the cases for high anxiety symptoms in the sample, while configurations LA1a, LA1b, LA2, and LA3 explained 63.3% of the cases for anxiety symptoms in the sample. The detailed configurational analysis results are shown in Table 9.

**Table 9:** Configurations for anxiety symptoms (fsQCA).

Variables	High AS		Low AS			
	HA1a	HA1b	LA1a	LA1b	LA2	LA3
SPB	●	●	—	—	⨂	⨂
PC	—	•	●	●	●	⊗
NC	●	●	•	—	•	⊗
SE	⨂	⨂	●	●	—	●
GSE	⊗	—	—	•	•	⊗
Consistency	0.865	0.857	0.844	0.854	0.841	0.946
Raw coverage	0.362	0.336	0.418	0.466	0.356	0.289
Unique coverage	0.093	0.067	0.033	0.058	0.027	0.092
Solution consistency	0.850		0.829			
Solution coverage	0.429		0.633			

*Note:* ●, core condition; •, peripheral condition; ⨂, absent as a core condition; ⊗, absent as a peripheral condition.

Abbreviations: AS, anxiety symptoms; GSE, general self‐efficacy; NC, negative coping; PC, positive coping; SE, self‐esteem; SPB, self‐perceived burden.

The fsQCA analysis reveals two major pathways leading to high anxiety symptoms and four configurational pathways for low anxiety symptoms.

The configuration of high anxiety shows high consistency. These two configurations are collectively categorized into a single pattern, namely, the SPB and PC co‐occurrence type. Both of them take high SPB, high NC, and low SE as the core combination.

The fsQCA analysis identifies four configurations associated with low anxiety symptoms, which can be summarized into three core protective patterns.

First is the resource‐sufficient type, corresponding to configurations LA1a and LA1b. This pattern is characterized by the simultaneous presence of PC and high‐SE as core features. Patients maintain positive coping styles and stable self‐worth. Even if there is a certain degree of PC (LA1a) or low GSE (LA1b), low anxiety can still be maintained as long as the core protective factors remain robust.

Second, is PC dominant type, corresponding to configuration LA2. Its main feature is the presence of PC as a core condition, accompanied by the absence of SPB. This pattern indicates that low anxiety levels can still be maintained as long as PC exists, and SPB is low even without high SE.

Third is a low SPB and high‐SE dominant type, corresponding to configuration LA3. This pattern is characterized by low SPB and high SE. The results suggest that patients can maintain low anxiety levels as long as they experience low SPB and high SE, even when they lack positive coping styles and GSE.

### 3.7. Robustness Analyses

The results of robustness analyses are summarized in the Supporting information. For high depression and anxiety symptoms, varying the frequency thresholds and anchors yielded results consistent with the primary solutions. Moreover, modifying the anchors led to a modest reduction in the solution consistency and coverage. For low depression and anxiety symptoms, the changes of the frequency thresholds and anchors both caused a slight difference in the specific configurational paths and elements. Among these paths, high SPB transformed into core or peripheral conditions, while the role of high PC as a core condition disappeared. The results indicated substitutability and complementarity between SPB and PC. Overall, the solutions from the principal analysis remained stable across all robustness analyses.

## 4. Discussion

Throughout the entire treatment spectrum, women with ovarian cancer exhibit markedly higher rates of depression and anxiety symptoms than their healthy counterparts [5]. In our study, 22.4% of the patients had mild or more severe depression symptoms, and 17.7% of people had light or more severe anxiety symptoms. Multiple interacting factors contribute to the emergence of depression and anxiety in patients with ovarian cancer—spanning genetic predispositions, health behaviors and lifestyle, the strength of social networks, and broader structural social factors [46, 47]. SPB and reliance on NC showed positive associations with depression and anxiety symptoms, whereas PC and higher SE were inversely related to these symptoms. GSE was inversely related to depression symptoms, but its association with anxiety symptoms was not statistically significant. Anxiety is more related to worries and fears about future uncertainties, while depression is more associated with negative past experiences and current low mood states [48]. Therefore, GSE may primarily influence an individual’s coping with current difficulties, with less impact on worries about future uncertainties in ovarian cancer patients.

Traditional net effect analysis focuses on estimating the independent effect of a single variable on health outcomes, which conflicts with the core concept of the social determinants of health, that multiple factors are combined together and work in conjunction through their joint actions [49].

FsQCA yielded three configurational paths leading to high depression symptoms; each configuration contained low SE alongside low GSE. The combination of low SE and low GSE with other conditions also emerged as core conditions in each configuration, which indicated that low SE and low GSE are essential components of the pathways associated with elevated depressive symptoms. SE is conceptualized as a psychological resource that, by fostering feelings of social acceptance and worth, helps regulate the terror elicited by awareness of mortality’s inevitability [50]. In this study, GSE is a belief that a person can use their abilities and skills to produce desired outcomes through their actions [51]. Low SE and GSE could work together so that patients could doubt their abilities and values when facing pressure or uncertainty in dealing with ovarian cancer [52].

In the configuration HD1, patients experience a significant psychological burden. The adverse stressor of cancer disrupts the everyday routines and planned activities of both patients and their caregivers [53]. Under the care of caregivers, patients are prone to experiencing the negative feeling of being a burden to others, which leads them to experience psychological distress and negative emotions [54, 55]. Hill et al. found that SPB can indirectly influence depression and anxiety symptoms in ovarian cancer patients through the effect of loneliness [56]. In the configuration HD2, NC emerged as one of the core conditions. The configuration further illustrates the connection between psychological resources and coping styles. The low levels of psychological resources will make patients more inclined to choose negative coping styles such as giving up and avoidance [57]. Such an association may coincide with individuals’ negative self‐assessment and diminished sense of life meaning, thereby generating pathways that are consistently observed among cases with high depression symptoms. In configuration HD3, attitudes of compromise and denial are not effective in dealing with the perceived burden [58]. Patients’ multiple concerns about their caregivers’ physical health, psychological well‐being, and financial situation may evolve into negative emotions such as guilt and depression symptoms [59].

The configuration for low depression symptoms falls into two categories. The first category is displayed as the configuration LD1, where PC, SE, and GSE exist as core conditions. Ovarian cancer patients with high‐SE and ‐GSE possess stronger psychological adaptability and are more likely to adopt a more proactive approach [60, 61] to dealing with the various challenges brought by cancer, thereby maintaining low levels of depression symptoms [62, 63]. LD2a and LD2b belong to the second category, both having a low perceived burden and high SE as core conditions. Prior studies indicate a significant inverse association between the SE and SPB. High SE can serve as a psychological resource for patients against the impact of stress [64]. In the configurations LD2a and LD2b, regardless of the coping style, low or high level, they were all peripheral conditions for low depression symptoms. When low SPB and high SE coexist in ovarian cancer patients, the association of coping styles with lower depression symptoms appeared attenuated and largely marginal. LD2b only showed the existence of SE as a condition, and the rest of the conditions were at a low level. This suggests that high SE is characteristic of configurations associated with low depression symptoms, particularly when SPB is absent or at low levels.

Configuration analysis obtained two paths, HA1a and HA1b, for high anxiety symptoms in ovarian cancer patients. Their core conditions were completely consistent, including SPB and NC and low SE. In configurations characterized by the absence of SE, patients facing high SPB tend to show fewer positive responses such as solving problems and seeking advice and more negative responses, alongside reduced stress alleviation. This pattern is characteristic of configurations associated with high anxiety symptoms and may reflect difficulties in regulating negative emotions. Patients with low SE tend to attribute stress events and negative feedback to their own shortcomings [48]. The high level of SPB and low SE boost patients’ motivation to engage in negative coping.

For low anxiety symptoms, fsQCA yielded four configuration paths belonging to three categories. The first category was the configurations LA1a and LA1b, which had two identical core conditions: high PC and SE. High SE enables individuals to accept their negative emotions without over‐criticizing themselves for short‐term failures or setbacks [65]. As a positive psychological resource, high SE can stimulate positive coping to help patients quickly recover from negative emotions and stressful situations. Sufficient coping resources encourage patients to use positive coping methods to help individuals build strong psychological resilience, and such configurations are associated with lower levels of anxiety.

The configuration LA2 took low SPB and high positive coping as core conditions, while the configuration LA3 took low SPB and high SE as core conditions. When the SPB of ovarian cancer patients is low, the presence of a positive coping style or high SE, along with appropriate levels of NC and GSE, is characteristic of configurations associated with low anxiety symptoms. In addition, the configuration LA3 was completely consistent with that of LD2b. The low SPB reduces the need for coping strategies and GSE, and the high SE provides a psychological buffer. At this time, patients may be more inclined to let nature take its course rather than be excessively concerned or anxious.

Based on the analysis results, patients with depression and anxiety were categorized into distinct types, and feasible clinical intervention strategies were proposed according to the configurational characteristics of each type.

For patients with the SPB‐dominant type presented in the high depression configuration, a common feature is the strong perception of being a burden to their families, accompanied by low self‐worth. We can intervene by targeting three key areas: reducing burden perception, enhancing self‐esteem, and decreasing negative coping. Through cognitive restructuring and enhancing positive interactions between patients and family members, patients can achieve more positive feedback within the family. Patients should help establish a strengths identification list and encourage them to practice self‐affirmation. Additionally, maladaptive coping styles should be addressed by discouraging avoidance and self‐blame while promoting active problem‐solving.

Among patients with high depression of the resource‐deficit type, personal coping resources are generally limited, and negative coping is pronounced. Recommendations include enhancing self‐esteem, improving coping strategies, and concurrently boosting self‐efficacy. When patients express low self‐efficacy through statements such as “I can’t do it” or “I’m incapable,” clinicians should share successful coping cases from patients in similar situations and accompany the patient in practicing coping skills.

High anxiety is characterized by the reciprocal reinforcement of SPB and negative coping. Intervention strategies for this population should prioritize the concurrent implementation of cognitive restructuring to alleviate the perceived burden and the reshaping of coping styles. Furthermore, fostering robust self‐esteem in patients constitutes an essential therapeutic target.

## 5. Strengths and Limitations

Drawing on a configurational approach and the Transactional Model of Stress and Coping. This study employed fsQCA to examine how ovarian cancer patients appraise stressful situations, offering a fresh lens for understanding and intervening in depression and anxiety symptoms and also helping to make up for the shortcomings of traditional research that focuses on simple linear relationships based on symmetry. The combinations of conditions for low depression or anxiety symptoms were not simply the opposite of those for high depression or anxiety symptoms. Substitutability and complementarity were observed among the various elements in the configuration. Thus, we cannot simply infer the configurational paths of depression or anxiety symptoms. Differentiated intervention measures should be implemented for patients with different mental health status and needs.

Although this study has made innovations based on previous research, there are still many limitations. First, the sample has certain limitations: the single‐center nature of this study and the recruitment of participants exclusively from Liaoning, China. The results may be influenced by region‐specific cultural factors, including social norms regarding illness expression, family‐based caregiving patterns, and structural features of the healthcare system. This constraint limits the generalizability and broader dissemination of our findings. Second, because all study variables were assessed via self‐report, the data may be susceptible to common method biases. The Harman single‐factor test confirmed that the common method bias was negligible. Then, some configurations in this study are supported by relatively few cases; some pathways may be exploratory in nature and should be interpreted with caution. Future research should employ larger samples or mixed methods to test their robustness. In addition, due to the study’s cross‐sectional nature, causal relationships between variables cannot be inferred. For the imputation of missing values, we used mean imputation, which may underestimate the variance of the variables and weaken the correlations among them. Future research may adopt more advanced methods, such as multiple imputation, to further validate the robustness of the findings. Also, large‐sample and multicenter comprehensive research with quantitative analyses and integration of time‐series QCA to further elucidate the trajectories of depression and anxiety symptoms in patients with ovarian cancer.

## 6. Conclusion

There are multiple configurational pathways for depression and anxiety symptoms by examining the combined effects of stress sources, coping styles, and coping resources, which contribute to an understanding of the complex mechanisms underlying mental health issues in ovarian cancer patients. The condition combinations for low depression and anxiety symptoms are not simply the opposite of those for high depression and anxiety symptoms; instead, they exhibit asymmetry. SPB and self‐esteem play a crucial role in the configuration pathways for both high/low depression and anxiety symptoms. The configuration allows caregivers and medical staff to implement targeted measures to reduce burden perception and negative coping, enhance positive coping and self‐esteem, considering their substitutability and complementarity.

## Author Contributions

**Chaoying Ma and Jiani He**: conceptualization, data curation, formal analysis, writing – original draft, writing – review and editing. **Li Liu**: investigation, writing – review and editing. **Yi Zhang**: conceptualization, investigation, methodology, supervision, writing – review and editing. **Hui Wu**: conceptualization, supervision, writing – review and editing. **Yajing Wang**, **Meizhu Pan, and Yuli Song**: project administration. **Zhichen Liu**, **Hui Guo, and Yiqun Xiao**: writing – review and editing.

## Funding

This study was funded by the China Postdoctoral Science Foundation (Project Number 2021M693915).

## Conflicts of Interest

The authors declare no conflicts of interest.

## Supporting Information

Additional supporting information can be found online in the Supporting Information section.

## Supporting information


**Supporting Information** In the supporting information, we present the robustness test results of the fsQCA.

## Data Availability

The authors confirm that the data supporting the findings are available from the corresponding author upon reasonable request.
