# Supplementary material for: Configurational Paths of Depression and Anxiety Symptoms in Ovarian Cancer Patients: A Fuzzy‐Set Qualitative Comparative Analysis
Source: Depress Anxiety. 2026 Jul 18;2026:6451069. doi: 10.1155/da/6451069 (PMC13379899; doi:10.1155/da/6451069)
Supplement: Supplementary file 1 — Supporting Information In the supporting information, we present the robustness test results of the fsQCA. [file DA-2026-6451069-s001.docx]

**Supplementary Information**

Results of configuration analysis for robustness testing.

Table S1: Summary of robustness tests

| Outcomes |  | Calibration anchors/  frequency thresholds | | Number of  configurations | Solution  consistency | Solution  coverage | Configuration differences |
| --- | --- | --- | --- | --- | --- | --- | --- |
| High DS | Main analysis  Changing frequency thresholds | | p5, p50, p95/4 | 3 | 0.894 | 0.567 | - |
|  |  |  | p5, p50, p95/5 | 3 | 0.894 | 0.567 | None |
|  | Changing calibration anchors | | p7.5, p50, p92.5/4 | 3 | 0.884 | 0.558 | None |
| Low DS | Main analysis | | p5, p50, p95/4 | 3 | 0.896 | 0.675 | - |
|  | Changing frequency thresholds | | p5, p50, p95/5 | 2 | 0.899 | 0.662 | Reduced 1 configuration |
|  | Changing calibration anchors | | p7.5, p50, p92.5/4 | 3 | 0.891 | 0.654 | None |
| High AS | Main analysis | | p10, p50, p90/4 | 2 | 0.850 | 0.429 | - |
|  | Changing frequency thresholds | | p10, p50, p90/5 | 2 | 0.850 | 0.429 | None |
|  | Changing calibration anchors | | p12.5, p50, p87.5/4 | 2 | 0.847 | 0.427 | None |
| Low AS | Main analysis | | p10, p50, p90/4 | 4 | 0.829 | 0.633 | - |
|  | Changing frequency thresholds | | p10, p50, p90/5 | 4 | 0.894 | 0.567 | High SPB as a peripheral condition once |
|  | Changing calibration anchors | | p12.5, p50, p87.5/4 | 4 | 0.824 | 0.621 | None |

Note: DS, depression symptom, AS, anxiety symptom, P5, 5th percentile, P7.5, 7.5th percentile, P10, 10th percentile, P12.5, 12.5th percentile, P50, 50th percentile, P87.5, 87.5th percentile, P90 90th percentile, P95 95th percentile.
